# A Rare Case of Hyperglycemic-Hemichorea in a Young Patient

**DOI:** 10.7759/cureus.8483

**Published:** 2020-06-07

**Authors:** Meraj Fatima, Mohsin Iqbal, Saira Abbas, Deepak Kumar, FNU Jitidhar

**Affiliations:** 1 Neurology, Dow International Medical College, Dow University Hospital, Dow University of Health Sciences, Karachi, PAK; 2 Internal Medicine, Dow University of Health Sciences, Karachi, PAK; 3 Internal Medicine, Dow University Hospital, Karachi, PAK; 4 Neurology, Dow University of Health Sciences, Karachi, PAK; 5 Internal Medicine: Nephrology, Jinnah Postgraduate Medical Center, Karachi, PAK; 6 Internal Medicine, Dow Medical College/ Civil Hospital, Karachi, PAK

**Keywords:** chorea, hyperglycemic hemichorea, chorea hyperglycemia basal ganglia syndrome

## Abstract

Chorea is an abnormal, nonrhythmic, and purposeless movement of limbs. There is a long list of diseases responsible for chorea; long-standing hyperglycemia can sometimes result in it, which typically manifests on one side of the body. MRI brain is an added diagnostic tool, which commonly shows hyperintense basal ganglia lesion on T1-weighted images. Chorea in the context of hyperglycemia is a reversible and infrequent occurrence, best managed with insulin and haloperidol combination therapy. Here, we discuss a patient with hyperglycemic-hemichorea, whose symptoms resolved completely within two months of taking insulin and haloperidol.

## Introduction

Chorea is a rapid, involuntary, abnormal, and irregular movement of limbs, which can involve trunk, head, and neck occasionally. There are a variety of conditions resulting in chorea: cerebrovascular insult, hormone replacement therapy, L-Dopa, Huntington’s disease, thyroid disorder, Wilson's disease, poststreptococcal Sydenham's chorea, and autoimmune conditions are some examples. But these disorders cause bilateral symptoms most of the time [1]. Hemichorea is an infrequent, but reversible presentation associated with hyperglycemia [2]. It can be treated with correction of hyperglycemic state within two to 28 days on average [3]. Here, we present a young patient with left-sided hemichorea, diagnosed with hyperglycemia as a causative factor.

## Case presentation

A 30-year-old South Asian male with no known co-morbid, presented with the complaints of involuntary, abrupt, and purposeless movement of left upper and lower limbs along with difficulty walking for two weeks, but could ambulate without assistance. These movements suppressed partially by rest, and disappeared completely during sleep but aggravated on performing tasks. The patient did not have urinary or stool incontinence. History of undocumented, unintentional weight loss was present, noticed due to the loosening of clothes. On detailed questioning, he denied a history of head trauma, tongue bite, unconsciousness, memory impairment, neuropsychiatric symptoms, fever, sore throat, heat intolerance, joints pain, oral ulcers, photosensitivity, or jaundice. Past medical, surgical, or history of blood transfusion was not significant. He declined the use of illicit drugs or any antipsychotic medications. His father was diabetic and hypertensive. There was no history of similar illness in family members. His sleep, appetite, bowel habits were normal, but noticed an increase in the frequency of passing urine lately.

Higher mental functions were intact. Mini-mental state examination revealed intact cognitive function. Pupils were bilaterally equal and reactive to light and no Kayser-Fleischer ring appreciated on naked eye examination. Cranial nerves were intact, muscle mass was normal, power five/five in all limbs. Deep tendon reflexes and the sensory system were intact, while planters were bilaterally flexors. Cerebellar signs were negative, whereas gait was abnormal due to hyperkinetic dance-like movements. Milk Maid grip sign and pronator sign were positive on the left side. We could not assess Romberg’s sign. All other systemic examinations were within normal limits.

**Figure 1:**
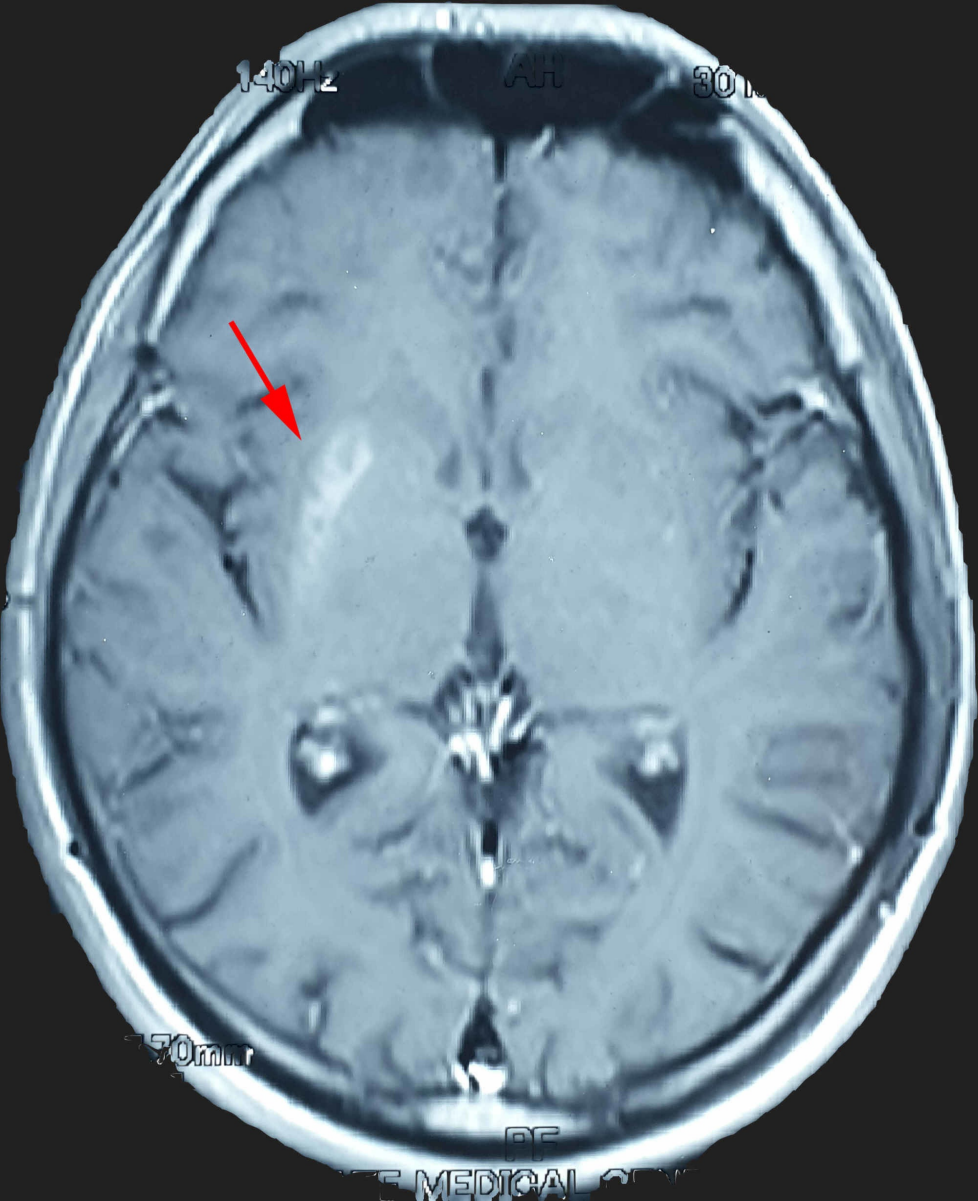
T1 Weighted MRI brain image. Arrow points toward right-sided basal ganglia lesion.

Random blood sugar was 453 mg/dL, while HbA1c 15.13% on patient presentation. We have presented blood/serum laboratory reports in Table 1. Arterial blood gas parameters were within normal limits on presentation. Urine analysis was insignificant with no ketones but positive glucosuria only. MRI brain with contrast showed right-sided basal ganglia hyperintense lesion in lentiform nucleus on T1 weighted images (Figure 1), but no ischemia or hemorrhages suggested. Hence, we diagnosed the patient with hyperglycemic-hemichorea (chorea-hyperglycemia-basal ganglia syndrome). We started our patient on insulin therapy along with haloperidol. His symptoms progressively improved and he was much better after 12 days of starting therapy. He improved completely after 55 days. Blood glucose levels remained under control and repeat HbA1c after two months was 8.24%.

**Table 1:** Blood investigations with results. MCV: mean corpuscular volume, ESR: erythrocyte sedimentation rate, HDL: high-density lipoprotein, LDL: low-density lipoprotein, VLDL: very low density lipoprotein, TSH: thyroid stimulating hormone, ANA: antinuclear antibody, ASO: antistreptolysin O, TIBC: total iron binding capacity, RBS: random blood sugar, HbA1c: glycated hemoglobin, SGPT: serum glutamic-pyruvic transaminase, SGOT: serum glutamic-oxaloacetic transaminase.

Blood/Serum investigation	Result	Reference range
Hemoglobin	11.8 g/dL	13.0-16.5 g/dL
MCV	76 fL	80-100 fL
White blood cells	10.3 x 10^3^/uL	4.0-11.0 x 10^3^/uL
Platelets	369 x 10^3^/uL	150-400 x 10^3^/uL
ESR	15 mm/h	Males: 0-15 mm/h
Urea	29.3 mg/dL	17-49 mg/dL
Creatinine	0.93 mg/dL	0.9-1.3 mg/dL
Sodium	130 mEq/L	136-146 mEq/L
Chloride	92 mEq/L	104-114 mEq/L
Bicarbonate	29 mEq/L	23-29 mEq/L
Potassium	4.1 mEq/L	3.5-5.1 mEq/L
Calcium	8.9 mg/dL	18-50 yrs: 8.8-10.2 mg/dL
Magnesium	2.0 mg/dL	Adult: 1.6-2.6 mg/dL
Phophorus	3.8 mg/dL	Adults: 2.7-4.5 mg/dL
C-Reactive protein	0.5 mg/dL	Less than 5 mg/dL
Total bilirubin	0.49 mg/dL	0.2-1.2 mg/dL
Direct bilirubin	0.19 mg/dL	0-0.3 mg/dL
Indirect bilirubin	0.3 mg/dL	0.25- 0.9 mg/dL
SGPT (ALT)	31 U/L	Less than 45 U/L
SGOT (AST)	29 U/L	Less than 35 U/L
Alkaline phosphatase	124 U/L	53-124 U/L
GGT	40 U/L	Less than 55 U/L
Triglyceride	234 mg/dL	Less than 150 mg/dL
Cholesterol	243 mg/dL	Less than 200 mg/dL
HDL	48 mg/dL	40-60 mg/dL
LDL	167 mg/dL	Less than 100 mg/dL optimal
VLDL	47 mg/dL	Less than 30 mg/dL
TSH	1.83 mIU/mL	Adult : 0.4-4.0 mIU/mL
Albumin	4.2 g/dL	3.4-5.0 g/dL
ANA	Negative	
Serum ceruloplasmin	22 mg/dL	20-35 mg/dL
ASO titer	Negative	Less than 200 IU/mL
Iron	78 ug/dL	Males : 59-158 ug/dL
TIBC	232 ug/dL	Males : 228-428 ug/dL
Ferritin	79.7 ng/mL	Males : 30-400 ng/mL
RBS ( on presentation )	453 mg/dL	80-140 mg/dL
HbA1c ( on presentation )	15.13%	Normal <5.7% Pre diabetics 5.7-6.4 % Diabetics >6.5%
HbA1c ( on follow up )	8.24%	Normal <5.7% Pre diabetics 5.7-6.4 % Diabetics >6.5%

## Discussion

Hyperglycemic-hemichorea was first reported in 1960 [4]. Hyperglycemia is the most common metabolic condition to cause chorea [5]. It is more common in females and the Asian population, but it does exist in other ethnicities [6-7]. The Asian population prevalence of this disorder is probably related to their genetics [5]. Older age is a risk factor, but the younger population has suffered it too [7], as in our patient.

Hyperglycemic-hemichorea involves an upper limb more than a lower limb. It manifests on one side of the body, but bilateral extremities can be affected, and rarely head and trunk are also involved [7]. Typically, it is diagnosed on clinical and radiographic findings: uncontrolled blood sugars with hemichorea, and lesions on basal ganglia on T1-weighted MRI brain images. Hyperintense lesion on T1 weighted MRI images is not specific to hyperglycemic-hemichorea and can occur in other central nervous system (CNS) pathologies, e.g. tuberous sclerosis, chronic hepato-encephalopathy, Tay-Sachs disease [8]. When radiographic findings are present, they are found on the contralateral side of patient symptoms, but imaging features are sometimes absent [5]. In bilateral limbs involvement, bilateral basal ganglia imaging lesions have been reported [9]. Lesion on MRI resolves in six months’ time after correction of hyperglycemia [10]. Moreover, Putamen is the most commonly involved site with or without the involvement of other regions of basal ganglia, but caudate and globus pallidus are almost never affected alone [9].

To date, the specific mechanism of the disease process is not clear. It has been thought to be multifactorial. Metabolic changes due to hyperglycemia resulting in a decrease in gamma aminobutyric acid (GABA) level are believed to cause this disorder, but it is not clear, why metabolic derangement affects one side of basal ganglia in most patients but not on both sides. Other theories suggest the cause being hyperglycemic hyperviscosity induced ischemia, petechial hemorrhages, calcification, and/or reactive astrocytosis [8-9, 11].

Management of hyperglycemic-hemichorea includes intravenous hydration and insulin therapy. Patients with disabling symptoms should be prescribed dopamine receptor blocking agent (e.g. haloperidol) [7]. Haloperidol is most effective while in some cases neuroleptics or antiseizure agents have shown partial effect or no effect at all in controlling symptoms [2]. In some studies, patients responded better with sugar control alone but hemichorea vanished completely with sugar control and haloperidol combination therapy [9].

## Conclusions

Young patients presenting with hemichorea should be looked for hyperglycemia as possible etiology for their ongoing illness; as chorea in the context of hyperglycemia is a rare, but reversible occurrence. Hyperglycemic-hemichorea can be better managed with insulin and haloperidol. Further studies are needed to best understand the pathophysiology, female predisposition, and more incidences in the Asian population.
